# Left ventricular apical aneurysm in Takayasu arteritis and chronic active Epstein–Barr virus infection

**DOI:** 10.1186/s43044-024-00540-z

**Published:** 2024-08-09

**Authors:** Karthik Raghuram, Arun Gopalakrishnan, Krishna Kumar Mohanan Nair, Narayanan Namboodiri, Ajitkumar Valaparambil

**Affiliations:** 1 Department of Cardiology, Sri Jayadeva Institute of Cardiovascular Sciences and Research, Bengaluru, Karnataka India; 2 Department of Cardiology, Sree Chitra Tirunal Institute for Medical Sciences and Technology, Thiruvananthapuram, Kerala 695011 India; 3 Department of Cardiology, KIMS Health, Thiruvananthapuram, Kerala 695011 India

**Keywords:** Aortoarteritis, Left ventricle, Left ventricular aneurysm, Takayasu arteritis, Epstein–Barr virus, Heart failure

## Abstract

**Background:**

Takayasu arteritis (TA) is a chronic inflammatory disease of unknown etiology characterized by a large vessel vasculitis involving the aorta and its branches. Myocardial involvement is extremely unusual in TA and is mostly in the form of myocarditis, ventricular hypertrophy, and ventricular dysfunction secondary to coronary ischemia. Submitral aneurysms have been reported in TA and has been attributed to the chronic inflammatory process in TA.

**Case presentation:**

We report a novel instance of left ventricular apical aneurysm in a 37-year-old lady with TA and normal epicardial coronaries. She was diagnosed with a left ventricular apical aneurysm, moderate aortic regurgitation, and moderate pericardial effusion. The coronary arteries were normal. The patient had concomitant chronic active Epstein–Barr virus infection complicating patient outcome.

**Conclusions:**

Left ventricular apical aneurysm with normal epicardial coronaries is a rare cause of heart failure in Takayasu arteritis. Concomitant chronic active Epstein–Barr virus infection can potentially accentuate the inflammatory process in Takayasu arteritis and complicate management and patient outcomes.

**Supplementary Information:**

The online version contains supplementary material available at 10.1186/s43044-024-00540-z.

## Background

Takayasu arteritis (TA) is a chronic inflammatory disease of large vessels, mainly involving the aorta and its branches. Valvular involvement and coronary artery lesions are the common cardiac manifestations of TA. Myocardial involvement is rare in TA. We report a rare case of left ventricular apical aneurysm in TA presenting with heart failure.

## Case presentation

A 37-year-old lady presented with complaints of acute onset dyspnea and chest discomfort for one day. The patient also gave a history of nasopharyngeal carcinoma managed with surgery and radiotherapy 20 years back.

At presentation, the patient was dyspneic with mild chest discomfort. On examination, she had sinus tachycardia (110 beats/minute), normal blood pressure, and jugular venous pressure elevated till the angle of the mandible. Precordial examination revealed cardiomegaly, left ventricular third heart sound, and an audible pericardial rub. Chest auscultation identified bilateral basal crackles. The electrocardiogram showed sinus tachycardia, normal QRS axis, T wave inversion noted in leads II, III, aVF, and V2-V6. Chest roentgenogram showed cardiomegaly with a cardiothoracic ratio of 0.65 and grade 1 pulmonary venous hypertension. Echocardiography revealed a left ventricular apical pseudoaneurysm with preserved thickness and contractility of the basal and mid-segments of the left ventricle. There were moderate aortic regurgitation and moderate circumferential pericardial effusion with no features of cardiac tamponade. Serial troponin T levels remained below the 99th centile, but the N-terminal pro-brain natriuretic peptide (NT-proBNP) was elevated (2140 pg/ml). Other blood investigations revealed anemia (hemoglobin—8.7 g/dl), elevated erythrocyte sedimentation rate (82 mm/1st hour), elevated C-reactive protein (88.92 mg/L), normal renal and liver function.

**Fig. 1:**
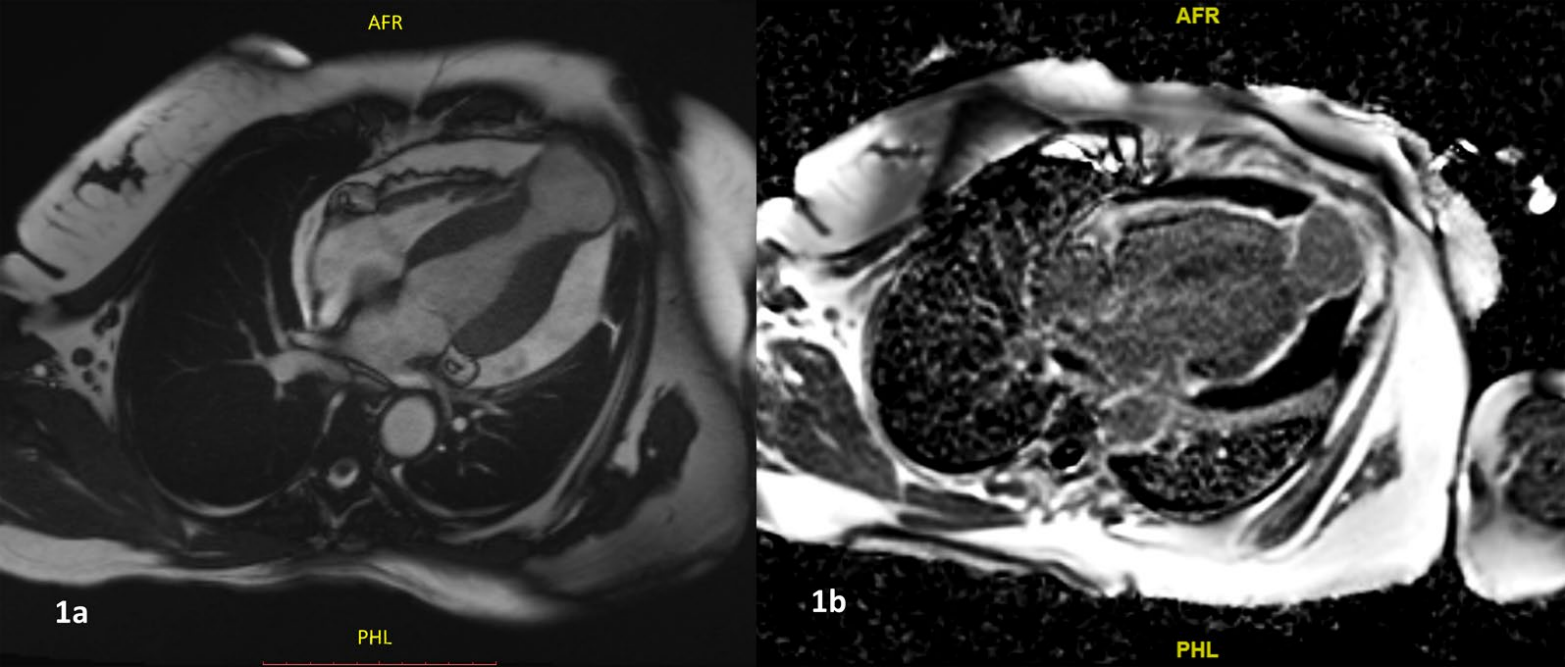
**a** Axial section of the cardiac MRI showing the left ventricular apical aneurysm and moderate circumferential pericardial effusion. Transmural late gadolinium enhancement of the aneurysmal segment is seen in (**b**)

**Fig. 2:**
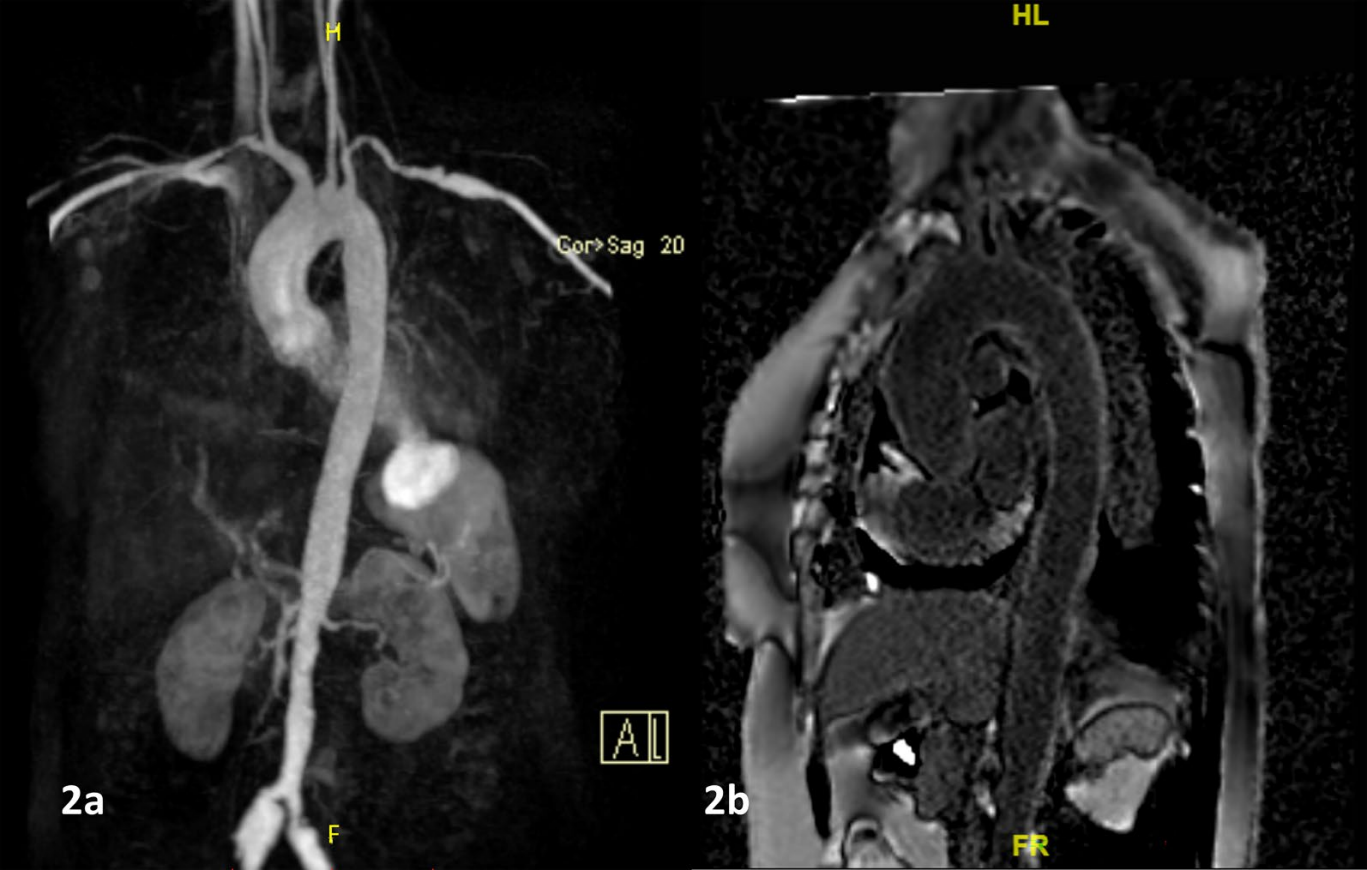
**a** Magnetic resonance angiogram showing stenosis of the proximal segments of bilateral subclavian arteries and infrarenal abdominal aorta. Fusiform dilatations of the distal left subclavian artery and bilateral common iliac arteries are also seen. **b** Late gadolinium enhancement of the aortic walls consistent with active aortoarteritis

Computed tomography showed a wide-necked aneurysm arising from the left ventricular apex with mural thrombus, mild circumferential wall thickening of aortic arch, descending thoracic aorta and proximal great vessels and irregular fusiform aneurysm of left axillary artery. Cardiac MRI confirmed apical pseudoaneurysm of the left ventricle and moderate circumferential pericardial effusion (Fig. 1a). The wall of the aneurysm was thinned out with transmural late gadolinium enhancement suggestive of fibrosis (Fig. 1b). The pericardial effusion was hypointense on T1 imaging suggestive of non-hemorrhagic effusion. Bilateral axillary fusiform aneurysms, outpouching from infra renal abdominal, bilateral common iliac fusiform aneurysms were noted consistent with aortoarteritis (Fig. 2a). Delayed gadolinium enhancement was noted involving the aortic arch, arch vessels, and descending thoracic aorta suggestive of disease activity (Fig. 2b).

Immunology workup reported negative for antinuclear antibody immunoblot profile and antinuclear cytoplasmic antibodies (pANCA and cANCA). Viral serological markers for HIV, HBsAg, HCV were negative. Epstein–Barr virus (EBV) serology was done. The reports were positive for the immunoglobulins IgG and IgM to both viral capsid antigen (anti-VCA) and anti-Epstein–Barr nuclear antigen (anti-EBNA).

A diagnosis of active Takayasu arteritis was made, and patient was started on immunomodulators after an immunology consultation. After control of disease activity, a coronary angiogram was done which revealed normal coronaries. The left ventricular angiogram showed left ventricular dysfunction and an apical aneurysm (Video 1). The patient was taken up for aneurysmectomy and surgical reconstruction (Dor’s procedure). However, the procedure was abandoned after identifying widespread inflammation intraoperatively with friable tissue. Patient was planned for staged re-operation and repair after a course of steroids and azathioprine. Unfortunately, the patient suffered a sudden cardiac arrest on the 10th postoperative day and could not be revived.

## Discussion

Takayasu arteritis (TA) is a chronic inflammatory disease of unknown etiology characterized by a large vessel vasculitis involving the aorta and its branches. The incidence of TA is 1–2 per million population and is predominantly described in young females in the second or third decade of life [1]. Unlike vascular disease, cardiac involvement is rarely a presenting feature in TA. While echocardiographic studies have noted that up to one-third of cases of TA can have cardiac valve lesions, advanced vascular pathology often dominates the clinical picture. Aortic valve incompetence is the most common valve affliction in TA followed by mitral regurgitation [2].

Myocardial involvement is extremely unusual in TA. Autopsy studies of TA have revealed hypertrophy of either or both ventricles, and evidence of myocarditis, besides ischemic changes secondary to coronary artery lesions [3]. Talwar et al. reported myocarditis as an important cause of heart failure in TA with pathological involvement in 83.3% of right ventricular endomyocardial biopsy specimens [4]. Systolic dysfunction of the left ventricle with global hypokinesia and normal epicardial coronaries is also reported in TA [5]. Myocardial ischemia related to coronary artery lesions, coronary arteritis, and aneurysms can also cause left ventricular dysfunction in TA often with postinfarction aneurysm formation [6, 7].

Apical aneurysms of the left ventricle with normal epicardial coronaries have not been reported in TA earlier to the best of our knowledge. Left ventricular pseudoaneurysms involving the basal portions can rarely present with mural thrombus formation and heart failure in TA [8]. Chronic focal inflammatory process in TA and healing with fibrosis could explain the myocardial thinning and late gadolinium enhancement in both these instances.

The implication of Epstein–Barr virus (EBV) seropositivity in the pathogenesis of left ventricular aneurysm in the index patient is unclear. EBV seropositivity has been reported in an immunocompetent patient with left ventricular apical aneurysm in primary cardiac diffuse large B-cell lymphoma [9]. EBV is known to cause lifelong latent infection in the lymph nodes of the host and reactivate during periods of physiological stress. EBV has also been rarely implicated in pathogenesis of autoimmune vasculitis. Murakami et al. reported an autopsy and histopathological examination of a case of large vessel vasculitis in a child infected with EBV [10]. They noted the detection of EBV DNA genome in diseased aortic tissue, and histopathological examination revealed moth-eaten appearance and destruction of medial elastic lamina.

Histopathological evaluation of myocardial tissue of the index patient could have shed light on whether chronic active EBV infection had a direct role in the pathogenesis of the ventricular apical aneurysm. However, widespread inflammation and advanced systemic illness forced the surgeon to back out.

## Conclusions

Left ventricular apical aneurysm with normal epicardial coronaries is a rare cause of heart failure in Takayasu arteritis. This is a potentially lethal complication and needs intensive anti-inflammatory treatment before surgical repair. Concomitant chronic active EBV infection can potentially accentuate the inflammatory process in Takayasu arteritis and complicate management and patient outcomes.

### Supplementary Information

Supplementary Material 1: Video 1: Left ventricular angiogram in right anterior oblique 30° view showing large left ventricular apical aneurysm.

## Data Availability

All data relevant are included in this published article [and its supplementary information files].

## Abbreviations

Anti-EBNA: Antibody to Epstein–Barr nuclear antigen
Anti-VCA: Antibody to viral capsid antigen (anti-VCA)
EBV: Epstein–Barr virus
MRI: Magnetic resonance imaging
TA: Takayasu arteritis
